# FunGene: the functional gene pipeline and repository

**DOI:** 10.3389/fmicb.2013.00291

**Published:** 2013-10-01

**Authors:** Jordan A. Fish, Benli Chai, Qiong Wang, Yanni Sun, C. Titus Brown, James M. Tiedje, James R. Cole

**Affiliations:** ^1^Center for Microbial Ecology, Michigan State UniversityEast Lansing, MI, USA; ^2^Department of Computer Science and Engineering, Michigan State UniversityEast Lansing, MI, USA; ^3^Department of Microbiology and Molecular Genetics, Michigan State UniversityEast Lansing, MI, USA; ^4^Department of Plant, Soil and Microbial Sciences, Michigan State UniversityEast Lansing, MI, USA

**Keywords:** microbial ecology, functional genes, amplification primers, phylogeny, biogeochemical cycles, amplicon analysis

## Abstract

Ribosomal RNA genes have become the standard molecular markers for microbial community analysis for good reasons, including universal occurrence in cellular organisms, availability of large databases, and ease of rRNA gene region amplification and analysis. As markers, however, rRNA genes have some significant limitations. The rRNA genes are often present in multiple copies, unlike most protein-coding genes. The slow rate of change in rRNA genes means that multiple species sometimes share identical 16S rRNA gene sequences, while many more species share identical sequences in the short 16S rRNA regions commonly analyzed. In addition, the genes involved in many important processes are not distributed in a phylogenetically coherent manner, potentially due to gene loss or horizontal gene transfer. While rRNA genes remain the most commonly used markers, key genes in ecologically important pathways, e.g., those involved in carbon and nitrogen cycling, can provide important insights into community composition and function not obtainable through rRNA analysis. However, working with ecofunctional gene data requires some tools beyond those required for rRNA analysis. To address this, our Functional Gene Pipeline and Repository (FunGene; http://fungene.cme.msu.edu/) offers databases of many common ecofunctional genes and proteins, as well as integrated tools that allow researchers to browse these collections and choose subsets for further analysis, build phylogenetic trees, test primers and probes for coverage, and download aligned sequences. Additional FunGene tools are specialized to process coding gene amplicon data. For example, FrameBot produces frameshift-corrected protein and DNA sequences from raw reads while finding the most closely related protein reference sequence. These tools can help provide better insight into microbial communities by directly studying key genes involved in important ecological processes.

## Introduction

Microbes are a major component of the earth's biosphere. They impact the earth's ecosystems through a diverse set of activities and constitute a major force in shaping both abiotic and biotic environments. Examining the prevalence and diversity of the genes involved in these important ecological processes—ecofunctional genes—can help us define the relationships between microbial populations and their environments. Amplicon-based analysis of 16S rRNA gene fragments has shown great utility in providing an overall picture of taxonomic diversity and has been used in a multitude of environments (see, e.g., Gilbert and Meyer, 2012). However, much of the microbial gene content, including pathways for important environmental activities, have been subject to multiple horizontal gene transfers (see, e.g., Dagan et al., 2008). Directly querying for specific functional genes in environmental samples, through qPCR and amplicon surveys, can provide information on the prevalence and diversity of key functional genes regardless of discontinuity between organism and gene phylogeny.

In contrast, those core protein-coding genes that are less subject to horizontal transfer and have gene phylogenies congruent with organism taxonomy can serve as important phylogenetic markers. These are often capable of resolving finer differences than the commonly used rRNA gene markers. Our concept of a microbial species is still evolving and a subject of debate, but the most commonly accepted ad hoc microbial species boundary has been 70% DNA-DNA re-association (Stackebrandt et al., 2002). Using this 70% re-association species definition, Stackebrandt and Ebers found that strains of the same species almost always share more than 99% full-length 16S rRNA gene identity, while different species can share identical 16S gene sequences (Stackebrandt and Ebers, 2006). The short 16S gene fragments commonly used in environmental amplicon analysis have even less resolving power. In contrast, the corresponding overall ortholog Average Amino Acid Identity (AAI; see list of abbreviations, Table 1) cutoff for a species is about 5% difference (Konstantinidis and Tiedje, 2005). This may vary for different marker genes, but in general protein-coding markers are capable of finer-grained resolution than rRNA genes (Santos and Ochman, 2004).

**Table 1:** **List of Abbreviations**.

AAI	Average Amino Acid Identity
BIOM	Biological Observation Matrix
ENA	European Nucleotide Archive
FGP	RDP Functional Gene Pipeline
FGR	RDP Functional Gene Repository
HMM	Hidden Markov Model
HMP	Human Microbiome Project
indel	insertion or deletion
IUPAC	International Union of Pure and Applied Chemistry
JNI	Java Native Interface
MID	Multiplex Identifier
MSA	Multiple Sequence Alignment
NEON	National Ecological Observatory Network
NGS	Next Generation Sequencing
OTU	Operational Taxonomic Unit
RDP	Ribosomal Database Project
rQ	Read Quality
SFF	Standard Flowgram Format
UPGMA	Unweighted Pair Group Method with Arithmetic Mean
WGS	Whole-Genome Shotgun

Several toolkits for analyzing 16S data are currently available; examples include the RDP's Pyrosequencing Pipeline (Cole et al., 2009), QIIME (Caporaso et al., 2010), CloVr (Angiuoli et al., 2011), mothur (Schloss et al., 2009), and VAMPS (http://vamps.mbl.edu/). However, while parts of published 16S pipelines can be reused in functional gene pipelines, functional gene analysis presents several challenges not seen in 16S rRNA analysis. Indel sequencing errors cause frameshifts that lead to garbled protein translations. Few tools offer the functional gene reference sets required for analysis. FunFrame (Weisman et al., 2013) is an R-based analysis pipeline for functional gene data, built on analysis tools including HMMFrame (Zhang and Sun, 2011) for frameshift correction and gene translation. However, FunFrame comes customized for only one gene, Cytochrome D1. Sequences for different functional gene families can be analyzed using a common set of tools; however, each functional gene requires its own set of reference sequences and requires different approaches tuned to take into account factors such as relative gene diversity and amplicon size. Protein databases such as Pfam (Punta et al., 2012), TIGERFAM (Haft et al., 2003), and Uniprot (Uniprot Consortium, 2010) contain well-curated protein family sequence sets, however, they mainly concentrate on protein not gene sequences. In addition, the protein families are often defined more broadly than optimal for ecological analysis, for example covering paralogous genes active on different substrates. None of these general protein databases provide the tools necessary for functional gene amplicon analysis.

The RDP's functional gene repository and analysis tools are available on two related websites. The FunGene Repository (FGR; http://fungene.cme.msu.edu/) is designed to help researchers explore publicly available sequences harvested from GenBank (Benson et al., 2004) and assigned to a specific collection of gene families. The FunGene Pipeline (FGP) houses tools for researchers to process and analyze their own functional gene sequencing data.

FGR employs a reference/model-based, comparative analysis strategy to build the reference database and help to study functional and phylogenetic diversities of specific gene families. This strategy relies on the use of HMMER3 (http://hmmer.org/) and Hidden Markov Models (HMM), the same tool used by the well-known Pfam for studying protein homology. HMMs are built from protein seed alignments, i.e., typically a small number of vetted, full-length representative sequences from each gene family aligned by hand or with a Multiple Sequence Alignment tool [MSA; e.g., ClustalW (Larkin et al., 2007)]. Using these models, more inclusive sequence sets are created for the FGR by extracting, classifying, and aligning sequences obtained from GenBank. A model-based alignment method was chosen for several reasons. With a model-based alignment, multiple sets of alignments from a single model can be merged and compared without re-aligning the sequence database. Model-based approaches allow for statistical classification of query reads as members/not members of the modeled gene family. Aligning sequences with an HMM scales linearly in time with the number of sequences and is fast enough to align, within reasonable time, hundreds of thousands to millions of sequences. With HMM-based alignments, the conservation statistics of amino acid residues, essential to protein structure and function, can be preserved. This alignment is then back-translated to provide a high-quality nucleotide alignment, allowing more accurate comparative analysis at the nucleotide level based on sequence homology.

Using FGP, libraries of sequence reads can be analyzed through either reference-based or unsupervised approaches after common initial processing steps. Reference-based approaches, such as the FrameBot frameshift correction and nearest neighbor tool (Wang et al., 2013) offered by FGP, require a set of representative sequences, which can be compiled using FGR. Reads are binned based on the nearest neighbor and associated distance. The unsupervised approach we offer involves clustering reads based on sequence identity using alignments generated by HMMER3. Either method can be used to compute various biodiversity metrics to characterize the phylogenetic and functional structures of communities. To accommodate the differences in analyses of sequences from different functional gene families, we have developed a reconfigurable pipeline of tools with a specific configuration for each supported functional gene family. Workflow configurations were developed together with researchers and made available via the FGP.

The FGR and FGP have been used in a number of gene-targeted metagenomic studies and we continue to improve these resources based on feedback from researchers. An incomplete list citing FGR and FGP includes projects studying microbial communities from various habitats as a reference database (Spain et al., 2007; Wertz et al., 2009; Bull et al., 2012; Lin et al., 2012; Fang et al., 2013), a primer designing/testing tool (Henry et al., 2006; Philippot and Hallin, 2006; Bru et al., 2007; Leigh et al., 2007; Stedtfeld et al., 2007; Jones et al., 2008, 2011, 2012; Stres and Murovec, 2008, 2009; Iwai et al., 2010; Oakley et al., 2010, 2012; Suenaga, 2012; Vital et al., 2013), and a data processing/analysis pipeline (Bragina et al., 2011, 2013; Grube et al., 2012).

## Materials and methods

### FunGene repository

Sequences in the FGR are selected from GenBank in a five-step process. First the files for the bacterial, plant, environmental divisions and Whole Genome Shotgun (WGS) data for the current GenBank release are downloaded. Then the release files are converted to the BioSeq XML format using the asn2all (ftp://ftp.ncbi.nih.gov/ncbi-asn1) tool available from the GenBank ftp site. Next the coding sequence (CDS) annotations are read from the BioSeq files. However, since the gene and product annotations on CDS regions are free text fields and not always reliable, we do not map CDS to gene families in the FGR directly via the annotations. Instead, every gene family contained in the FGR has an associated HMM. These HMMs were built using HMMER3 from seed sequences selected as representatives by researchers interested in the particular gene family and from selected gene families obtained from Pfam. HMMER3 is used to scan the protein translation of every CDS record and default significance cutoffs are used to filter insignificant hits. Each translated GenBank CDS is scored with each HMM, and those with a significant hit to one or more FGR gene family's HMM are recorded in the FGR database. In addition to the protein sequence, the nucleotide sequence, bibliographic reference (if present), protein and nucleotide accession numbers, organism name, and description are also stored in the FGR database. When a new version of a record is released from GenBank, the existing version in the FGR database is replaced.

A Java Native Interface (JNI) wrapper around the HMMER3 scanning pipeline was developed in order to tightly integrate HMMER3 with the FGR release pipeline. The JNI wrapper is available as part of the RDP Alignment Tools package (Table 2). The JNI wrapper trades higher memory usage for faster running time by storing all HMMs in memory instead of rereading models for every query sequence.

**Table 2:** **Names and sites for all first-party tools used in the FunGene Pipeline**.

**Tool name**	**Available from**
Full pipeline scripts	http://github.com/rdpstaff/fungene_pipeline
RDPTools	http://github.com/rdpstaff/RDPTools
Initial process	http://github.com/rdpstaff/SeqFilters
Defined community analysis	http://github.com/rdpstaff/AlignmentTools
Dereplicator	See mcClust
FrameBot	http://github.com/rdpstaff/Framebot
mcClust	http://github.com/rdpstaff/Clustering
Rarefaction/Diversity measures	http://github.com/rdpstaff/AbundanceStats

The FGR currently contains 77 gene families organized into seven categories: Antibiotic resistance, Biodegradation, Biogeochemical Cycles, Metal Cycling, Phylogenetic Markers, Plant Pathogenicity, and “Other” for gene families not in the listed categories. FGR is intended to tap community efforts to expand its database. New gene families are added with each release and researchers are invited to work with the RDP to get new gene families incorporated into the FGR.

### FunGene pipeline

The FGP consists of a set of tools, along with reference files and parameters for each gene offered and Python scripts that coordinate running the individual tools that make up the pipeline. A Java EE5 Web Application powers the FGP website. All of the tools we developed and incorporated in the FGP are available under open source licenses from the RDPStaff GitHub page, http://github.com/rdpstaff/ (Table 2). GitHub is a website for hosting software in git repositories. The RDPTools repository contains instructions for downloading and building the other tools using git, GNU Make and Apache Ant. In addition, individual tool repositories are available on GitHub for those wishing to modify the code for a specific tool. Many of the downloadable FGP tools have additional options not available in the online version (see the README file in each tool package). All third-party tools incorporated in the FGP are freely available from their original sources (Table 3). Configuration options for the FGP pipeline are explained in more detail in the included README file. In addition to the full pipeline, FGP tools are also offered in a modular fashion so that researchers can substitute other tools for individual processing steps. For instance a researcher can substitute another chimera checking tool or MSA tool.

**Table 3:** **Names and sites for all third-party tools used in the FunGene Pipeline**.

**Tool name**	**Available from**
USEARCH 6.0 (UCHIME)	http://www.drive5.com/usearch/
HMMER3	http://hmmer.janelia.org/

#### Alignment

HMMs are included for all functional gene families supported by the FGP. Protein sequences are aligned with hmmalign from HMMER3. Sequence alignments are generated with the “—allcol” option which ensures alignments across samples are comparable.

#### Clustering

FGP uses the mcClust (http://github.com/rdpstaff/Clustering) clustering tool. mcClust is our implementation of a proposed single round memory-constrained clustering algorithm (Loewenstein et al., 2008). In this algorithm the distance between a pair of sequences is referred to as a thin edge and the collection of thin edges between every pair of sequences in two clusters is a thick edge. The algorithm takes as input a sorted list of thin edges; our implementation computes and sorts the distances on disk using either a single threaded algorithm, or a Hadoop (v0.18) map-reduce algorithm.

Each thin edge is processed by updating the appropriate thick edge weights. For complete linkage clustering, a thick edge's weight is the largest thin edge's weight seen thus far. A pair of clusters is merged when all thin edges between them have been seen.

For average linkage clustering, a thick edge's weight is the running average of the seen thin edges' weights. Unlike complete linkage clustering, where every thin edge between two clusters must be seen in order to know the weight of the thick edge connecting the two clusters, in average linkage clustering, the weight of the thick edge can be bounded. The weight of every thick edge is the average of all the thin edge weights between the two clusters. If a weight of zero is substituted for the unseen edge weights, a lower bound on the weight of the thick edge is obtained. If instead a weight of one is substituted, an upper bound is obtained. These bounds can be tightened by utilizing the fact that thin edges are processed in sorted order. The last seen thin edge weight can be used instead of zero to obtain a tighter lower bound, since all remaining thin edges must have weight greater than or equal to the last thin edge weight. A tight upper bound can be obtained by tracking the largest distance between any pair of sequences during the distance calculation step and using that value in the upper bound calculation. A pair of clusters is merged when the upper bound on weight of the thick edge corresponding to the last read thin edge is less than the smallest lower bound on any other thick edge's weight.

In the average linkage version of the algorithm, if the total memory available is exhausted before all distances have been read, the remaining distances are scanned, filling out thick edges between all the current clusters, and then clustering is resumed. This distance scanning can be done in one pass (our implementation of the single pass algorithm proposed by Loewenstein et al.) or in multiple passes over the distances (as in the multipass version presented by Loewenstein et al.).

The online tool provides complete linkage clustering only, but the downloadable version also supports single and average linkage algorithms. Multiple sequence files can be clustered together, where each file is treated as an individual sample or where all files are treated as a single sample, or a sample mapping file can be supplied to identify the sample from which every sequence came. Cluster files can be converted into formats suitable for loading into common statistical tools such as R (R Core Team, 2012) and estimateS (http://viceroy.eeb.uconn.edu/estimates/).

#### Defined community analysis

The Defined Community Analysis tool consists of two parts: the CompareErrorType program written in Java and the parseErrorAnalysis.py script written in Python. Both are available as part of the AlignmentTools package (Table 2). The CompareErrorType tool implements the standard Needleman-Wunsch algorithm (Needleman and Wunsch, 1970). CompareErrorType computes a global alignment between the read and each of the reference sequences. The pairwise alignment producing the highest alignment score is used to identify the source organism, tabulate substitutions, indels, and (optionally) associated quality scores. The CompareErrorType tool outputs the best pairwise alignment, the substitution data, indel data, and read quality scores to separate files. These data files are in turn processed by the parseErrorAnalysis.py script to summarize the types of errors found in the input reads.

#### Read quality score

The rQ score (read Q score), defined as rQ = −10× log(E), where E, the average predicted error rate for the read, is calculated as

$$E=\frac{1}{n}\sum_{i=1}^{n}10^{-}\frac{Q_{i}}{10}$$

from the per-base Q scores (Phred quality scores) provided by the sequencer base-calling software, where n is the number of bases in the read.

### Defined community composition

The *nifH* defined community contains three organisms: *Desulfitobacterium hafniense* DCB-2, *Nostoc* sp. PCC 7120, and *Burkholderia xenovorans* LB400. The genomic DNA was mixed together and amplified using the Poly primers (Poly et al., 2001) with one barcode (Wang et al., 2013) to produce sample NIFH. This defined community contains six *nifH* and *nifH*-like genes. The butyrate kinase (*buk*) defined community contains genomic DNA from five strains: *Clostridium perfringens* ATCC 13124, *C. difficile* 630, *C. acetobutylicum* ATCC 824, *Bacillus licheniformis* 14580, and *Bacteroidetes thetaiotaomicron* E50. All the strains contain a single *buk* gene, except *C. acetobutylicum* ATCC 824, which contains two copies. Samples BUK1 and BUK2 were each prepared separately. The *buk* defined community was PCR amplified in three reactions using three barcoded forward and three reverse primers as described by Vital (Vital et al., 2013). The three barcoded *buk* amplifications were then combined together. Sample NIFH and Sample BUK1 were sequenced on the 454 GS FLX Titanium platform at the Michigan State University Research Technology Support Facility (http://www.rtsf.msu.edu). Sample BUK2 was sequenced on the 454 GS Junior platform and obtained from Christopher Radek of Michigan State University. Sequencer base calling software version 1.1.03 was used for all three runs. We chose these three data sets because they were sequenced at different times and each represents different error characteristics. These sequences have been submitted to ENA Short Read Archive (http://www.ebi.ac.uk/ena/) under accession numbers PRJEB4229 and PRJEB4242.

## Tool descriptions

### FunGene repository

The FGR website provides an interface to interactively explore sequences and associated metadata for genes chosen for their utility in microbial community analysis. FGR currently (release 7.3) maintains a collection of 77 gene families in seven categories. Most of these genes are for key steps in important environmental processes. Many were chosen in collaboration with microbial ecologists and we continue to expand the numbers and categories of genes based on interests of the research community. New FGR releases occur bi-monthly to coincide with GenBank releases. For each release, new candidate CDS for the gene families are extracted and classified using protein profile HMMs.

The FGR organizes gene families by function. Detailed information is provided for each family, including sequence annotations, publication references, homology measures, and alignment views. Researchers can evaluate FGR sequences for a single family, as a selected subset, or as individual sequences. Researchers are able to explore the homology space of a gene family to help perform tasks such as correlating functional and phylogenetic diversities to disentangle orthologous from paralogous gene sequences, developing and assessing amplification primers, and compiling vetted reference sequence sets. Such reference sets can be used for refining HMMs, with FGP tools for frameshift detection/correction and for nearest neighbor assignment.

Gene family pages are accessible from the main page, each presents records of protein and nucleotide CDS matching an HMM that define one gene family. Researchers can set specific selection criteria for subsets of records to be rendered using the combination of filter options on sequence length, percentage of the HMM covered, and HMM matching score. The HMM for each gene family can be downloaded via a link on the gene family page.

The top of each page contains the gene symbol, total number of sequence records in this family, and HMM length. Data for each sequence in a gene family are presented in table format with up to 12 columns, described in Table 4, depending on display options. The table can be sorted on any column by clicking the column header. Records used to build the protein HMM for the gene family are highlighted in pink, making it easy to visually determine if the seed set could be improved, and for easy inclusion of the seeds in sequence subsets for further analysis.

**Table 4:** **Data columns on gene family pages**.

**Heading**	**Action**
[+]	Click to view the protein sequence aligned to HMM and reference consensus sequence
Select	Check to mark the selection for analysis (selections are saved in the researcher's session and are not lost when navigating to a new page)
Score	The HMM alignment score in bits saved (the higher the score, the higher the probability this sequence is a member of this gene family)
New_Hit	Marker for sequence records new to the current release
Environmental	Marker for non-cultured, environmental samples
Prot_Accno	GenBank protein accession number (also links to the actual protein record in GenBank or FASTA format depending on the researcher's current display settings)
Nuc_Accno	GenBank nucleotide accession number (also links to the actual nucleotide record in GenBank or FASTA format depending on the researcher's current display settings)
Organism	Name of source organism as annotated in the GenBank record
Definition	Gene and product as annotated in the GenBank record
Reference	Publication from GenBank record, links to NCBI PubMed live records when available
Size	Protein length (number of amino acids, proxy for completeness of the sequence)
HMM_Coverage	How completely the sequence covers the model's length (measured by the percentage of HMM positions to which the sequence is aligned, can be used to filter out partial sequences and poor HMM matches)

“Display Options” opens a page with options related to the current session view, including types of sequences displayed (environmental, isolates, or both), columns displayed, default sort column, sequences per page, and default sequence record format (GenBank or FASTA). “Show/Hide Filter Options” allows filtering displayed sequences by HMM score, protein length, and HMM coverage. The filtering criteria for each gene family are saved separately allowing researchers to navigate between gene families without losing their filter settings.

Sequence selections can be modified by using the links located at the top of the page: (1) Select the Entire Page, (2) Select All Sequences, (3) Deselect All Sequences, and (4) Select Seed Sequences. The sequences selected are dependent on the current filtering criteria. Individual records can be selected/deselected using the checkbox in the corresponding row of the table. A sequence counter at the top of the page shows the total number of sequences selected for the current gene family. Selections are saved for each gene family separately allowing researchers to navigate between gene families without losing their selections.

“Begin Analysis” opens a panel with options: (1) download selected sequences; (2) build a phylogenetic tree using Tree Builder; (3) test primers using Probe Match.

The Download panel provides options for download of either protein or nucleotide sequences in either aligned or unaligned FASTA or PHYLIP formats to allow users flexibility in additional analysis. FGR's alignments can be used for further analysis using third-party tools.

Tree Builder provides rapid phylogenetic tree reconstruction for selections of 4–200 sequences using the Approximate-Maximum-Likelihood method of FASTTREE (Price et al., 2010) from the choice of either protein or nucleotide alignment. The tree is displayed in a Java applet that allows interactive exploratory manipulations, such as selecting nodes, and swapping branches. Additional viewing options, as well as download options, are described on the tree page. Any selection made to the tree can be used to update the sequence selections for that gene family for further analysis using the FGR tools or for download.

Probe Match (Myers, 1999; Cole et al., 2005) performs a search against the selected nucleotide sequences (subjects) for matches to the entered oligonucleotide sequence (query). Standard IUPAC characters are allowed if “Allow ambiguity matches” is checked. The strand orientation of the subjects has to be specified. “Probe targets plus strand” should be checked for a reverse primer, and left unchecked for a forward primer. “Maximum distance” box is used to specify the total number of allowed differences (substitutions and indels) in the priming/probing region.

Probe Match returns results summary and search parameters. A table of individual hits contains six columns: (1) “Selection”—check to select the sequence; (2) “Detail”—protein accession number; (3) “Distance”—number of differences; (4) “Target/Probe”—the query aligned with the matching subject sequence region and differences marked in red; (5) “Definition”—gene and product as annotated in the original GenBank record; (6) “Organism”—name of the source organism as annotated in the original GenBank record. Within this result page, sequences can be selected/deselected as a whole through the “Select All/Deselect All” button or individually through the checkbox. Similar to the gene detail page, sequences selected here are available for additional analysis or download.

### FunGene pipeline

The FGP allows researchers to analyze their own functional gene sequence data. The FGP has reference sets for a subset of the gene families available in the FGR but can be used with any reference set (for any gene family) a researcher has created using FGR or other resources. The FGP contains a set of tools for processing and performing unsupervised analyses and reference-based analyses on functional gene data.

Many tools offered through the FGP can take up to several hours to run with large sequence datasets; the FGP was designed with this in mind. Most tools in FGP require an email address for the job to be submitted. This email address is only used to send a link to the result archive. The progress of a job can also be monitored in real-time on the job status page. This page shows the current processing step and is refreshed every 30 s until the job is completed. Upon completion the result archive will automatically start downloading. Researchers can optionally specify a job name that will be included in the results email and be the name of the archive containing the result files. The FGP automatically dereplicates uploaded sequence files to collapse duplicate sequences into a single sequence. After all processing for a job is complete, the resulting sequence files are then “re-replicated” and the final sequence files are placed in the “filtered_sequences” directory.

The FGP accepts compressed files for upload and supports the Gzip, Tar, Zip, and bzip2 formats. All FGP downloads are TAR-formatted archives compressed with Gzip that can be opened natively on OS X and Linux. On windows, several third-party tools exist for opening these types of archives, a common program being 7zip.

The tools are offered in a modular fashion allowing researchers to choose the appropriate ones based on their needs, and to assemble analysis procedures using our tools along with other third-party tools for individual processing steps. For instance, a researcher might cluster samples from two different analyses using the memory-efficient, complete-linkage algorithm implemented in mcClust. Or, a researcher might want to use a different chimera check tool other than UCHIME to identify chimeric sequences.

The FGP offers a “pipeline” where researchers can assemble a set of analysis tools to process a nucleotide sequence file, filter chimeric sequences, translate the nucleotide sequences, align, and cluster the protein sequences and additionally run the optional cluster file analysis tools. By using the pipeline, instead of running the individual tools, researchers can avoid uploading and downloading all the intermediate result files and, instead, get one result archive containing all the files generated during processing.

### Initial process tool

**Figure 1:**
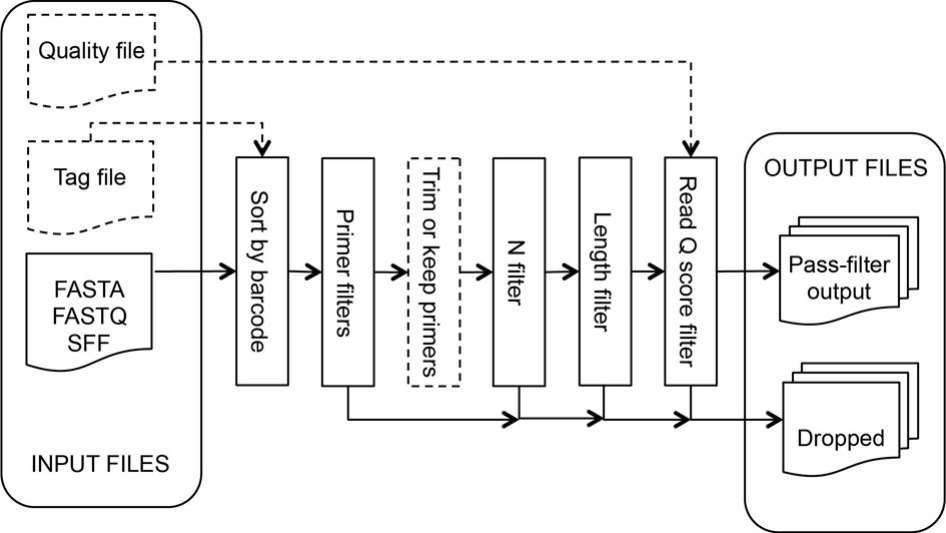
**Inputs, outputs, and the filters applied, in order, by Initial Process**.

This tool takes read files produced by the sequencer software, separates the reads by sample for multiplexed runs, and discards reads not passing researcher-specified quality filter values. If a tag [also known as barcode or Multiplex Identifier (MID)] file is specified, input sequences are sorted by tag before processing. PCR primers are identified and the read is trimmed up to or including the sequencing primer. The sequencing primer can optionally be left on reads; this can be beneficial when working with very short amplicons. This process, illustrated in Figure 1, discards sequences failing the following filters (in order): (1) number of differences to the forward amplification primer (Forward Primer filter), (2) number of differences to the reverse amplification primer (Reverse Primer filter), (3) number of ambiguous bases (N filter), (4) length of the sequence (Length filter), and (5) estimated read error rate (Read Q Score filter).

#### Inputs

Read files in FASTA, FASTQ, and SFF format, or a compressed file containing multiple sequence files. If a FASTA file is uploaded, researchers can optionally supply a separate quality file in QUAL format, a FASTA-like format where the sequence string is replaced by the integer quality scores for a sequence, each separated by a space. Researchers can optionally upload a Tag File containing a tab-delimited list of tag, sample ID pairs. Researchers supply a list of forward primers using standard IUPAC ambiguity characters for degenerate positions and an optional list of reverse primers. At least one sequence file and one forward primer are required.

The Forward Primer filter searches the read for the best match to one of the specified forward primers, allowing up to a specified number of differences (default: 2). If a match is not found, the read is discarded. The 5′ end of the read, up to and including the match, is then removed unless the Keep Primer option is specified.

The Reverse Primer filter is similar to the Forward Primer filter. This filter searches for the best match to one of the specified reverse primers up to a user-specified number of differences (default: 1). However, since in practice sequencers often do not read to the end of the template, this filter then attempts to find a reverse primer prefix at the end of the read using a relaxed matching heuristic that weights a match as 1, a difference as −6, and requires a final weight of 6 or more. No differences are allowed if the user specifies 0 differences. If a match is not found, the read is discarded. The 3′ portion of the read starting at the match is then removed unless the “Keep Primer” option is specified. If no reverse primer sequences are provided, this filter is not applied.

The N filter discards reads containing more uncalled bases, “N”s, than the allowance specified by the researcher (default: 0).

The Length filter discards reads that are shorter than the researcher-specified minimum length (default: 150) after applying the above filters. The measured length will not include the primer portion unless the “Keep Primer” option is specified.

The Read Q Score filter discards reads with a predicted error rate greater than specified. Input as rQ (default: 20; See methods), where the predicted error rate is 10^(− *rQ*/10)^.

#### Outputs

A compressed file of a directory containing a set of subdirectories, one for each tag plus a NoTag subdirectory for reads that did not match any of the input tags. Each directory contains trimmed sequences and quality files in FASTA format, quality and length statistics in text and graphical formats, a text file listing the reads that failed a filtering step and which filter it failed, a text file listing the best matching primer and the edit distance for each read, and a summary file with information such as the number of reads matching the tag and passing each filter. The trimmed sequence and quality files are ready for use in additional analysis steps. The other output files can be useful in troubleshooting sequencing problems such as excessive numbers of short reads, high errors in primer regions or low sequence quality.

### Dereplicator tool

The dereplicator tool is designed to “de-duplicate” one or more input sequence files to speed up computationally intensive tools by avoiding performing the same analysis on exact duplicate sequence strings. The dereplicator tool works by collapsing identical sequence strings down to a single unique entry, keeping track of the IDs of the duplicate sequence strings in an ID mapping file, and for each sequence, the input file name in a sample mapping file. The explode mappings tool takes a de-duplicated sequence file and associated ID and sample mapping to reinflate to a fully replicated sequence file. To improve the speed, many of the web tools use the dereplicator tool internally. These tools return the intermediate de-duplicated output files along with the fully replicated results.

#### Inputs

Nucleotide sequence file(s).

#### Outputs

(1) A FASTA file of the unique sequences from the input sequence file, (2) an ID mapping file, with each line consisting of a list of sequence IDs for identical sequences in the input sequence file, the first of which is used in the FASTA output file, (3) a sample mapping file containing a list of sequence ID, sample name pairs for each sequence.

### Chimera check

The Chimera Check tool uses UCHIME from the USEARCH package (Edgar et al., 2011) to detect chimeric sequences from an amplicon sequence file. Note that UCHIME is run in *de novo* mode, which relies on abundance information contained in the original sequence read files. Sequence files in which read abundances have been altered are not appropriate in this mode.

#### Inputs

Nucleotide sequence file(s).

#### Outputs

(1) A directory named “chimera_check” which contains the raw UCHIME results for unique sequences only, (2) a directory named “chimera_filtered_sequences” containing FASTA files for the non-chimeric sequences, (3) a directory named “filtered_mapping” containing the ID mapping and sample mapping files for the non-chimeric sequences.

### Defined community analysis tool

This tool compares reads to the set of known sequences for amplification targets in the amplified DNA. It determines the numbers of amplicons corresponding to each amplification target and the numbers and types of errors present in the reads. Three types of errors are measured: nucleotide insertion, deletion, and substitution. All of these measures can be used to assess the quality of a sequencing run, and tune processing and quality filtering parameters for analysis of experimental samples.

#### Inputs

A nucleotide sequence file containing the input reads in FASTA/FASTQ format, an optional quality file in QUAL format if a FASTA sequence file is used, and a FASTA file containing the known amplification target reference sequences. The amplicon reads and the reference sequences should cover the identical region. It may be convenient to trim the reference sequences to match the exact region being sequenced by using the Initial Process tool with corresponding forward and reverse primers.

#### Outputs

A compressed file containing the following results: (1) a text file with pairwise alignments between each read and its closest reference sequence, (2) a tab-delimited file containing detailed information about each substitution error, including the read ID, closest reference sequence ID, substitution position in the alignment, the expected base and observed base, (3) a tab-delimited file containing detailed information about each insertion and deletion error, including read ID, closest reference sequence ID, indel position in the alignment, expected homopolymer length, observed homopolymer length, indel base, indel position in the read, indel position in the reference, and Q score of the extra base for insertions, (4) a tab-delimited file containing rQ scores for each sequence if quality file provided, and (5) a summary file including total substitutions and indels, the number of reads per target reference, and errors summarized by type, reference and rQ score. This summary can be input into a spreadsheet program to produce summary charts. A Python script can be downloaded from the RDPStaff GitHub repository (http://github.com/rdpstaff/fungene_pipeline/) to produce the summary from the other four output files with additional options or on a subset of the reads to avoid having to reprocess an entire defined community to tweak input parameters.

### FrameBot

FrameBot (Wang et al., 2013) is a frameshift-correction and nearest neighbor assignment tool. The online version of FrameBot has reference sequence sets compiled for all the genes included in the FGP through collaboration with researchers. Researchers can alternatively supply their own reference set when processing sequences with FrameBot.

#### Inputs

Nucleotide sequence file(s) and either a Gene Name chosen from the pre-configured reference sets or a file with target protein reference sequences. The aligned protein length cutoff option (default: 80) discards sequences that are below the length cutoff. The percent protein identity cutoff (default: 0.4) discards the reads with lower protein identity. These options can be used to filter out non-target reads.

#### Outputs

The compressed output file, when expanded, contains: (1) a directory named “framebot” containing the raw FrameBot results for unique sequences only, a text file containing the pairwise alignment of the passed sequences to the nearest neighbor in the reference set, a FASTA file with frameshift-corrected protein sequences, a FASTA file with frameshift-corrected nucleotide sequences, a text file containing the pairwise alignment of the failed sequences, and a FASTA file with the failed nucleotide sequences; (2) a directory named “filtered_sequences” containing FASTA files of the frameshift-corrected protein sequences; (3) a directory named “filtered_mapping” containing the ID mapping and the sample mapping files for the sequences passing FrameBot.

### Aligner

The alignment tool uses HMMER3 to align protein sequences to a model for a gene family. Sequence alignments attempt to place homologous residues for the different sequences into the same alignment column, enabling downstream analysis, e.g., phylogenetic reconstruction and gene family modeling. Alignments produced by the FGP Aligner are comparable to each other, for the same gene family, however, the files cannot be concatenated to create a valid alignment file. Instead the alignments must be merged; this is done automatically by FGP tools when a researcher uploads multiple aligned sequence files.

#### Inputs

FASTA formatted protein sequence file(s). The researcher must also select the gene family with which to align the uploaded sequences.

#### Outputs

The resulting alignment file is contained in the result archive. The alignment file is in FASTA format and contains one additional meta sequence: #=GC_RF. This sequence defines the columns of the MSA that are comparable (non-insert) positions and must be present in all alignment files uploaded to FGP tools.

### mcClust

The mcClust tool computes the pairwise uncorrected distances between sequences and then performs complete linkage hierarchical clustering on the resulting distance matrix. Clustering is used in OTU analysis and is typically an intermediate step to obtain an OTU by sample matrix for statistical analysis. mcClust reports all clusters up to a specified distance cutoff at a set interval (step). A sample mapping file can be provided to override mcClust's default behavior of treating each input file as a distinct sample. The sample mapping file should contain the same number of sequence IDs as the sequences uploaded. Each line contains a sequence ID and the name of the sample it comes from separated by a space, the sample name should not contain spaces. The output cluster file can be converted to BIOM (McDonald et al., 2012) format using a tool available in the mcClust package. Also the cluster file can be converted to a tab delimited OTU abundance matrix format usable by many statistical tools via the Cluster to R-Format tool.

#### Inputs

Aligned sequence file(s), distance cutoff (default: 0.5), step size (default: 0.01), and optional sample mapping file.

#### Outputs

A cluster file in the RDP cluster file format (.clust). The first line contains a list of the sample names and the second the number of sequences from each sample. Then, at each cutoff, the distance is listed along with the total number of clusters at that distance followed by the detailed cluster membership. Each cluster is one line per sample with the same cluster ID, followed by the name of the sample, number of sequences in the cluster, and finally a list of all sequence IDs in that cluster. Samples with no sequences in a given cluster are not listed in the cluster file to save space.

### Representative sequence tool

The representative sequence tool selects a sequence from each cluster in a cluster file as the representative for that cluster. Researchers can then use this single sequence as a proxy for the cluster in downstream analysis and apply the results to all sequences in the cluster. Care must be used to ensure results transferred from a representative sequence to a cluster are not overly specific for the distance between the sequences in the clusters. For instance, at a 10% protein distance, all sequences in a cluster may not be from the same species. The representative sequence is selected by the method of least squares.

#### Inputs

A cluster file in RDP cluster file format (.clust) and sequence alignment file as well as the clustering distance at which to find representative sequences.

#### Outputs

The resulting archive contains a FASTA file with the aligned representative sequence for each cluster; the cluster is listed in the description field for each sequence in the FASTA file.

### Diversity estimates

The FGP offers diversity estimate tools for computing several alpha and beta diversity estimates and rarefaction curves from a cluster file.

Rarefaction estimates OTU richness as a function of sampling effort and is commonly used to assess whether the sequencing depth is sufficient to capture most diversity. The output archive file contains graphs of the rarefaction curves for each sample in the uploaded cluster file and text files containing the data used to generate the rarefaction curves.

Shannon and Chao1 Indices are two alpha diversity estimates. Shannon Index is used to assess both richness and evenness of OTUs in the sample, while Chao1 estimates the number of OTUs in the sample adjusted for unseen diversity. The result archive contains a text file listing alpha diversity estimates for every sample at each distance cutoff in the uploaded cluster file.

Jaccard and Sørensen Indices are two beta diversity measures of the similarity between samples. In FGP, these two indices are computed with Chao abundance corrections (Chao et al., 2006). This tool requires that two or more samples be represented in the input cluster file, i.e., a cluster file derived from two or more alignment files. The result archive contains the sample similarity matrices, one file per distance measure (Jaccard or Sørensen) and per distance cutoff. A dendrogram generated from the UPGMA clustering of the sample similarity matrices as well as a heatmap representing the sample similarities are included for each distance cutoff. These images are generated using R with a script, which is also included in the download archive and can be modified to regenerate the images.

## Use case

Here we use the three defined community data sets to demonstrate use of the FGP analysis tools on NGS sequencing data. We concentrate on samples amplified from genomic DNA of a set of known organisms (a “mock community”), as it is good practice to include one or more such control samples with each sequencer run. This helps troubleshoot any problems that may arise during both amplification and sequencing steps, and can provide important quality control information for the sequencer run. These data can be used to optimize the processing parameters for the particular amplicon and, more importantly, help validate comparisons between samples sequenced on different sequencer runs, as the quality of sequencing data can vary between different runs and between minor vender platform updates.

A well-constructed defined community should contain organisms for which whole genome sequences are available and where copies of the targeted gene(s) are found across multiple organisms representing the range of diversity expected in the experimental samples. Inclusion of organisms with closely related paralogs can help define the primer specificity, as can inclusion of targets with varying degrees of differences to the primers. For example, the Human Microbiome Project (HMP) developed a defined community consisting of genomic DNA from 22 organisms as proxy for human associated communities (http://www.hmpdacc.org).

First we examine amplification of a 321 bp region of the *nifH* gene coding for nitrogenase reductase from a defined community made up of three organisms known to fix nitrogen. Although a community of only three members, this example illustrates several important points in a control. The chosen organisms come from three different bacterial phyla. The genome of one, *Desulfitobacterium hafniense* DCB-2, in addition to the *nifH* gene, contains three copies of *nifH*-like genes with different degrees of primer match that could possibly co-amplify with *nifH*. Another member, *Nostoc* sp. 7120, contains two *nifH* gene copies with different degrees of primer match. Careful selection of PCR conditions could be used to optimize *nifH* target amplification while limiting amplification of non-target *nifH*-like genes (although beyond the scope of this work). The *nifH* control amplification was multiplexed with 10 other amplified *nifH* samples and sequenced on the 454 GS FLX Titanium platform.

### Initial processing

We first processed the sequencing run containing the NIFH defined community sample using the Initial Processing tool to sort reads by tag using the default quality filtering parameters: maximum forward primer differences 2, reverse primer differences 1, no Ns, minimum length 300, and minimum rQ score 20. Some of these choices are pragmatic; the chance of identifying the wrong read region as a primer match, even when allowing for two differences, is low for primers of average length and degeneracy. The presence of Ns (uncalled bases) in primer regions has been linked to low quality sequences (Huse et al., 2007). The length filter setting should normally reflect what is known about the expected amplicon length distribution. For the vast majority of *nifH* genes, the amplified gene fragment will be about 321 bp long. Using a slightly lower cutoff will still remove aberrant short amplicons, such as primer-dimers. We applied a moderate rQ score filter of 20. Using an rQ cutoff can help remove low quality sequences, but a high cutoff, as was suggested (Kunin et al., 2010), can lead to taxon specific bias, as shown below.

From the summary output file, 5790 reads matched the tag, and 5505 (95%) passed all filter steps. There were 249 reads that failed either the Forward Primer or Reverse Primer filters, 23 that failed the 0 N filter, nine that were shorter than 300 bases, and four with rQ score less than 20. These filters removed abnormal reads—those from aberrant PCR products, for example. In our experience, it's not uncommon for 5–10% of reads to be filtered from an otherwise good run. If there are more than this, the Initial Process output files can be used to examine the reason each read failed to help troubleshoot the amplification and sequencing steps.

At this stage we can use the FrameBot tool to detect and correct frameshifts, and to determine the source of each read. If this were a sample of unknowns, FrameBot could be used to find the closest matching reference sequence to each read, along with the percent identity. FrameBot requires a set of reference sequences. Here we used a set of *nifH* gene sequences derived from the FGR, making sure to include the seven *nifH* and *nifH*-like genes carried by the three defined community members (Table 5). The Poly primers we used do not perfectly match the reference organisms with the exception of *B. xenovorans* LB400. [A recent review of *nifH* primers found several other primer pairs with better overall coverage (Gaby and Buckley, 2012)]. Only three genes, one from each organism, produced significant reads. It is notoriously hard to quantify and mix equimolar amounts of genomic DNAs, but discrepancies between input molar ratios of genomic DNAs and numbers of amplicon sequence reads have been noted before (Jumpstart Consortium Human Microbiome Project Data Generation Working Group, 2012). The results between the four *D. hafniense* genes and between the two *Nostoc* sp. genes correlate with the number of differences to the PCR primer regions on the genome sequences. Note that this is not because the resulting amplicons were rejected by the primer filters. After the initial rounds of primer hybridization and extension, the amplicon primer regions match the sequence of, and are physically derived from, the synthetic oligonucleotide primers, not the genome sequence.

**Table 5:** **Number of amplicon reads that passed Initial Process assigned to each NIFH defined community organism**.

**Gene ID**	**Strain**	**Primer differences**		**Reads**
		**Forward**	**Reverse**	
ACL19109.1	*Desulfitobacterium hafniense* DCB-2	1	0	3784
ACL19859.1	*Desulfitobacterium hafniense* DCB-2	2	2	4
ACL19409.1	*Desulfitobacterium hafniense* DCB-2	4	6	0
ACL19588.1	*Desulfitobacterium hafniense* DCB-2	1	3	0
BAB73411.1	*Nostoc* sp. 7120	1	1	405
BAB72831.1	*Nostoc* sp. 7120	1	2	2^*^
YP_553849.1	*Burkholderia xenovorans* LB400	0	0	1310

* *These two sequences were poor matches at the amino acid level. Further testing found that both were chimeric sequences between Nostoc* sp. 7120 *and B. xenovorans* LB400.

### Defined community error analysis

**Figure 2:**
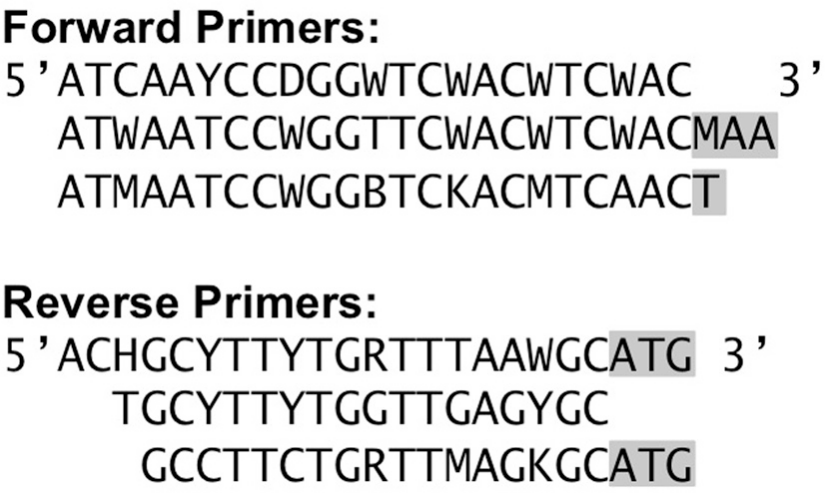
***buk* amplification primers (Vital et al., 2013)**. Bases in gray were not used as the primer sequence inputs in the Initial Process in order to capture the same gene region from each read.

It can be very difficult to assess the quality of a sequencer run from the sequencer output statistics or even from the number and types of anomalies flagged by the Initial Process tool. Also, the effects of the Initial Process filters can vary depending on the specific amplicon and the overall quality of the sequencer run, so examining the effects of the Initial Process options on a defined community sample can help optimize the analysis parameters used for the experimental samples. Here we used our Defined Community Analysis tool to measure error types and rates, and two third-party tools to detect chimeric and contaminant reads. In addition to the NIFH-defined community sample used above, we will analyze two samples of *buk* gene fragments (coding for butyrate kinase) amplified from a second defined community. To better match the diversity of known *buk* genes, three primer sets were used and the products mixed after amplification. The amplicon region between the three forward primers and three reverse primers does not start or end at the same position (off by 1, or 3). We truncated the primer sequences submitted for Initial Process such that they ended at the same relative position (Figure 2). The BUK1 and BUK2 samples were amplified separately and each was multiplexed with additional samples for sequencing. The BUK1 sample was sequenced using the 454 FLX Titanium platform, while the BUK2 sample was sequenced on the 454 GS Junior platform (Table 6).

**Table 6:** **Number of amplicon reads that passed Initial Process assigned to each BUK defined community organism**.

**Gene ID**	**Strain**	**Primer differences**		**Match BUK1 reads**	**Match BUK2 reads**
		**Forward**	**Rev.**		
NP_811465.1	*B. thetaiotaomicron* Strain E50	3	3	0	0
YP_079736.1	*B. licheniformis* 14580	0	0	749	363
NP_349675.1	*C. acetobutylicum* ATCC 824	0	0	68	229
NP_348286.1	*C. acetobutylicum* ATCC 824 2nd	0	1	38	22
YP_001086582.1	*C. difficile* 630	0	1	1183	82
YP_697036.1	*C. perfringens* ATCC 13124	0	0	244	1110

To examine the errors in all three defined community samples, we first passed the raw read data through the Initial Processing tool using less stringent filtering than above: maximum forward primer differences 2, reverse primer differences 2, no Ns, minimum length 300, and rQ score of 0. This allowed more error-prone sequences to pass so we could explore the relationship of the number of reverse primer differences and quality score to sequence errors. In this example, we focus on the impact of the rQ Score filter, to show the potential unintended consequences of too stringent quality filtering, and the Reverse Primer filter because errors tend to occur more frequently in the distal end of the reads (Gilles et al., 2011) hence errors in the reverse primer may be an indication of high distal sequence error rates. But the tools provide the necessary information to allow users to examine the effects of the Forward Primer filter and N filter, if desired.

Errors were quantified using the Define Community Analysis tool, which compares the nucleotide reads to the corresponding amplicon region from the defined community organisms. The summary files for the three samples were then loaded into Excel spreadsheets to calculate the error rates. A small number of reads had a relatively high percentage of differences to the known defined community. These usually represent small numbers of chimeric or contaminant reads. The sensitivity of the amplicon PCR sequencing method is one of its strengths, but it also means that it is common for small amounts of contaminants to be represented in the resulting reads. If the numbers are small, they will have little effect on experimental samples and, if desired, can be excluded when the summary files are regenerated using an option with the Python script described in the Tool Description section. For these examples, we verified that some of the reads with ≥10% differences to the closest community sequence were chimeric, using UCHIME (Edgar et al., 2011), and using BLASTN via the NCBI BLAST website (Altschul et al., 1990) we verified most others were closely related to *nifH* genes from organisms not in the defined community and thus likely contaminants (Table 7). These reads were excluded from further analysis. The overall error rate per base was 0.13% for NIFH, 0.41% for BUK1, and 1.2% for BUK2. The rates for the first two compare favorably with the claimed error rate for the 454 Titanium platform (1.07%; Gilles et al., 2011), while the error rate for BUK2 is similar to the claimed error rate for the junior platform (1.88%; Loman et al., 2012). The number of errors (nucleotide insertions, deletions, and substitutions) for each sequence ranged from 0 to 39. For the NIFH sample, 76.3% of reads perfectly matched the defined community, while only 28.1% and 3% of the BUK1 and BUK2 reads, respectively, matched the defined community perfectly. The *buk* amplicon is longer than the *nifH* amplicon, 420 vs. 321 bp, explaining some of the error rate differences; but as the two BUK samples illustrate, there can be a large difference in error rates between runs, even when both runs have error rates better than the expected platform performance.

**Table 7:** **Summary information for the three defined community samples**.

**Sample**	**Reads passed filtering**	**Average length**	**Error rate per base**	**Reads with no errors (%)**	**Reads with indels (%)**	**Chimeras**	**Contaminants**
NIFH	5509	321	0.13	76.3	14.3	25	1
BUK1	2334	420	0.41	28.1	54.6	2	0
BUK2	2206	421	1.2	3.0	85.9	5	11

To examine the effects of the two filters in detail, the result files were parsed using the Python script to produce additional summary files for various subsets of the reads. For example, by excluding reads with 0 difference to the reverse primer, we obtained the summary result for reads with 1 or 2 differences to the reverse primer.

**Figure 3:**
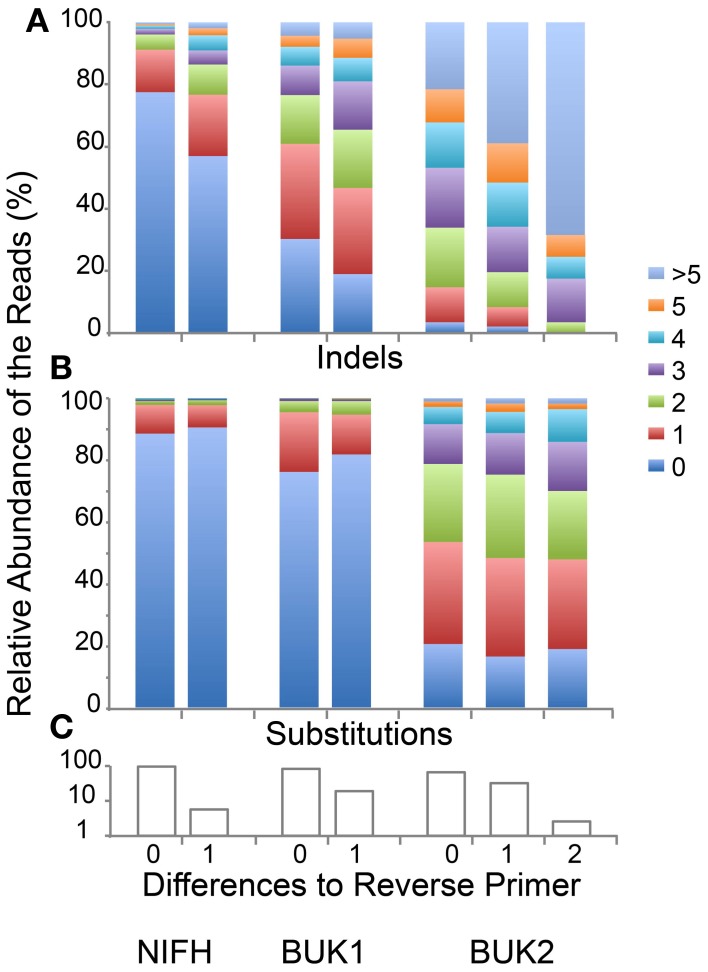
**Indels and substitutions varying by differences to reverse primer. (A)** Fraction of reads with the given number of indels for each reverse primer difference. **(B)** Fraction of reads with the given numbers of substitutions for each reverse primer difference. **(C)** Fraction of total reads with exactly the specified number of differences to the reverse primer. Eight BUK1, 57 BUK2, and no NIFH reads had two primer differences (NIFH and BUK1 with two differences were not shown).

Changing the Initial Process filters will modify both the number and quality of sequences passing the filters. To illustrate this point, we re-ran the three samples allowing only 0, 1, or 2 differences to the Reverse Primer (Figure 3). Only a few additional sequences passed with 2 differences for the BUK1 and BUK2 samples and none for the NIFH sample. In all three cases, reads with no reverse primer differences averaged fewer errors, even though the primer regions were not included in the error count. This is largely due to increasing indel errors with increasing primer differences. Substitution errors differed only slightly with primer differences. Whether it makes sense to discard reads with reverse primer errors would depend on the experimental goals. If the reads are going to be matched to references, e.g., using FrameBot, additional random errors are unlikely to change the identity of the nearest matching reference. For the NIFH sample, less than 6% of reads have reverse primer differences, while 35% of the BUK2 reads have such differences. About half BUK2 reads have multiple substitutions, independent of primer difference. For unsupervised approaches, such as clustering, even a small number of reads with high level of substitutions will greatly inflate the diversity estimates (Reeder and Knight, 2009; Kunin et al., 2010). Using the Defined Community Analysis tool can help flag this problem.

**Figure 4:**
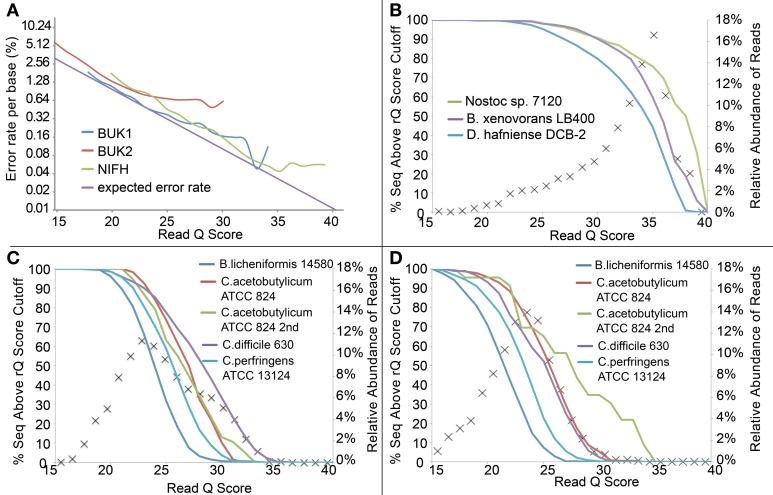
**(A)** Expected error rate and observed error rate per base (nucleotide insertions, deletions, and substitutions) from the three defined community data sets. Only data points with more than 5 reads are shown. The expected error rate was calculated based on the formula in the Materials and Methods. **(B–D)** Percentage of sequences matching each defined community organism by read Q score for Samples NIFH, BUK1, and BUK2, respectively. Symbol “×” represents the relative abundance of reads at each read Q score, and solid lines represent the percent of sequences above the read Q score cutoff from each sample.

Similarly, we modified the rQ Score cutoff to discard those reads expected to have more sequencing errors. On average, the actual error rates tracked the expected rates reasonably well for the NIFH and BUK1 sample, but for the BUK2 sample, lower expected error rates did not translate to fewer errors for the better scoring reads (Figure 4A). Of more interest was the distribution of read quality scores between different taxa (Figures 4B–D). The effect was not pronounced for the NIFH sample, only becoming noticeable above an rQ score of 25, but the effect was much larger for the BUK1 and especially the BUK2 sample, where differences were apparent between taxa at rQ scores below 20. Although judicious use of a quality cutoff can remove a few aberrant high-error sequences from an otherwise high quality sequencer run, applying a stringent quality filter is unlikely to salvage a marginal sequencer run.

## Summary

Microbial processes are interdependent and underpinned by the community metabolic network, made up of interactive activities from individual organisms in complex modes. The genes coding for these activities form the fundamental framework of such systems and help define the potential relationships between the microbial populations and the environment. Studying such functional genes, their products, and their spatial-temporal patterns provides a direct approach for developing dynamic community models. The FGR provides collections of such genes in an interactive platform, while the FGP offers a suite of tools for functional gene amplicon processing and analysis. Together they enable the key steps in functional gene-based microbial community analysis, from target selection and primer analysis to amplicon processing and ecological discovery.

### Conflict of interest statement

The authors declare that the research was conducted in the absence of any commercial or financial relationships that could be construed as a potential conflict of interest.

## References

[B1] Altschul S. F. Gish W. Miller W. Myers E. W. Lipman D. J. (1990). Basic local alignment search tool. J. Mol. Biol. 215, 403–410 10.1016/S0022-2836(05)80360-2 2231712

[B2] Angiuoli S. V. Matalka M. Gussman A. Galens K. Vangala M. Riley D. R. (2011). CloVR: a virtual machine for automated and portable sequence analysis from the desktop using cloud computing. BMC Bioinform. 12:356 10.1186/1471-2105-12-356 21878105 PMC3228541

[B3] Benson D. A. Karsch-Mizrachi I. Lipman D. J. Ostell J. Wheeler D. L. (2004). GenBank: update. Nucl. Acids Res. 1, D23–D26 10.1093/nar/gkh045 14681350 PMC308779

[B4] Bragina A. Berg C. Müller H. Moser D. Berg G. (2013). Insights into functional bacterial diversity and its effects on Alpine bog ecosystem functioning. Sci. Rep. 3:1955 10.1038/srep01955 23739741 PMC6504810

[B5] Bragina A. Maier S. Berg C. Müller H. Chobot V. Hadacek F. (2011). Similar diversity of alphaproteobacteria and nitrogenase gene amplicons on two related sphagnum mosses. Front. Microbiol. 1:275 10.3389/fmicb.2011.00275 22294982 PMC3261640

[B6] Bru D. Sarr A. Philippot L. (2007). Relative abundances of proteobacterial membrane-bound and periplasmic nitrate reductases in selected environments. Appl. Environ. Microbiol. 73, 5971–5974 10.1128/AEM.00643-07 17630306 PMC2074903

[B7] Bull M. J. Marchesi J. R. Vandamme P. Plummer S. Mahenthiralingam E. (2012). Minimum taxonomic criteria for bacterial genome sequence depositions and announcements. J. Microbiol. Methods 89, 18–21 10.1016/j.mimet.2012.02.008 22366464

[B8] Caporaso J. G. Kuczynski J. Stombaugh J. Bittinger K. Bushman F. D. Costello E. K. (2010). QIIME allows analysis of high-throughput community sequencing data. Nat. Methods 7, 335–336 10.1038/nmeth.f.303 20383131 PMC3156573

[B9] Chao A. Chazdon R. L. Colwell R. K. Shen T.-J. (2006). Abundance-based similarity indices and their estimation when there are unseen species in samples. Biometrics 62, 361–371 10.1111/j.1541-0420.2005.00489.x 16918900

[B10] Cole J. R. Chai B. Farris R. J. Wang Q. Kulam S. A. McGarrell D. M. (2005). The Ribosomal Database Project (RDP-II): sequences and tools for high-throughput rRNA analysis. Nucl. Acids Res. 33, D294–D296 10.1093/nar/gki038 15608200 PMC539992

[B11] Cole J. R. Wang Q. Cardenas E. Fish J. Chai B. Farris R. J. (2009). The ribosomal database project: improved alignments and new tools for rRNA analysis. Nucl. Acids Res. 37, D141–D145 10.1093/nar/gkn879 19004872 PMC2686447

[B12] Dagan T. Artzy-Randrup Y. Martin W. (2008). Modular networks and cumulative impact of lateral transfer in prokaryote genome evolution. Proc. Natl. Acad. Sci. U.S.A. 105, 10039–10044 10.1073/pnas.0800679105 18632554 PMC2474566

[B13] Edgar R. C. Haas B. J. Clemente J. C. Quince C. Knight R. (2011). UCHIME improves sensitivity and speed of chimera detection. Bioinformatics 27, 2194–2200 10.1093/bioinformatics/btr381 21700674 PMC3150044

[B14] Fang H. Cai L. Yu Y. Zhang T. (2013). Metagenomic analysis reveals the prevalence of biodegradation genes for organic pollutants in activated sludge. Bioresour. Technol. 129, 209–218 10.1016/j.biortech.2012.11.054 23247148

[B15] Gaby J. C. Buckley D. H. (2012). A comprehensive evaluation of PCR primers to amplify the nifH gene of nitrogenase. PLoS ONE 7:e42149 10.1371/journal.pone.0042149 22848735 PMC3405036

[B16] Gilbert J. A. Meyer F. (2012). Modeling the earth microbiome. ASM Microbe 7, 64–69 22465599

[B17] Gilles A. Meglécz E. Pech N. Ferreira S. Malausa T. Martin J.-F. (2011). Accuracy and quality assessment of 454 GS-FLX Titanium pyrosequencing. BMC Genomics 12:245 10.1186/1471-2164-12-245 21592414 PMC3116506

[B18] Grube M. Köberl M. Lackner S. Berg C. Berg G. (2012). Host–parasite interaction and microbiome response: effects of fungal infections on the bacterial community of the Alpine lichen Solorina crocea. FEMS Microbiol. Ecol. 82, 472–481 10.1111/j.1574-6941.2012.01425.x 22671536

[B19] Haft D. H. Selengut J. D. White O. (2003). The TIGRFAMs database of protein families. Nucl. Acids Res. 31, 371–373 10.1093/nar/gkg128 12520025 PMC165575

[B20] Henry S. Bru D. Stres B. Hallet S. Philippot L. (2006). Quantitative detection of the nosZ gene, encoding nitrous oxide reductase, and comparison of the abundances of 16S rRNA, narG, nirK, and nosZ genes in soils. Appl. Environ. Microbiol. 72, 5181 10.1128/AEM.00231-06 16885263 PMC1538733

[B21] Huse S. M. Huber J. A. Morrison H. G. Sogin M. L. Welch D. M. (2007). Accuracy and quality of massively parallel DNA pyrosequencing. Genome Biol. 8, R143 10.1186/gb-2007-8-7-r143 17659080 PMC2323236

[B22] Iwai S. Chai B. Sul W. J. Cole J. R. Hashsham S. A. Tiedje J. M. (2010). Gene-targeted-metagenomics reveals extensive diversity of aromatic dioxygenase genes in the environment. ISME J. 4, 279–285 10.1038/ismej.2009.104 19776767 PMC2808446

[B23] Jones C. M. Graf D. R. H. Bru D. Philippot L. Hallin S. (2012). The unaccounted yet abundant nitrous oxide-reducing microbial community: a potential nitrous oxide sink. ISME J. 7, 417–426 10.1038/ismej.2012.125 23151640 PMC3554408

[B24] Jones C. M. Stres B. Rosenquist M. Hallin S. (2008). Phylogenetic analysis of nitrite, nitric oxide, and nitrous oxide respiratory enzymes reveal a complex evolutionary history for denitrification. Mol. Biol. Evol. 25, 1955–1966 10.1093/molbev/msn146 18614527

[B25] Jones C. M. Welsh A. Throbäck I. N. Dörsch P. Bakken L. R. Hallin S. (2011). Phenotypic and genotypic heterogeneity among closely related soil-borne N2- and N2O-producing Bacillus isolates harboring the nosZ gene. FEMS Microbiol. Ecol. 76, 541–552 10.1111/j.1574-6941.2011.01071.x 21348884

[B26] Jumpstart Consortium Human Microbiome Project Data Generation Working Group. (2012). Evaluation of 16S rDNA-based community profiling for human microbiome research. PLoS ONE 7:e39315 10.1371/journal.pone.0039315 22720093 PMC3374619

[B27] Konstantinidis K. T. Tiedje J. M. (2005). Towards a genome-based taxonomy for prokaryotes. J. Bacteriol. 187, 6258–6264 10.1128/JB.187.18.6258-6264.2005 16159757 PMC1236649

[B28] Kunin V. Engelbrektson A. Ochman H. Hugenholtz P. (2010). Wrinkles in the rare biosphere: pyrosequencing errors can lead to artificial inflation of diversity estimates. Environ. Microbiol. 12, 118–123 10.1111/j.1462-2920.2009.02051.x 19725865

[B29] Larkin M. A. Blackshields G. Brown N. P. Chenna R. McGettigan P. A. McWilliam H. (2007). Clustal W and Clustal X version 2.0. Bioinformatics 23, 2947–2948 10.1093/bioinformatics/btm404 17846036

[B30] Leigh M. B. Pellizari V. H. Uhlík O. Sutka R. Rodrigues J. Ostrom N. E. (2007). Biphenyl-utilizing bacteria and their functional genes in a pine root zone contaminated with polychlorinated biphenyls (PCBs). ISME J. 1, 134–148 10.1038/ismej.2007.26 18043623

[B31] Lin X. Kennedy D. Peacock A. McKinley J. Resch C. T. Fredrickson J. (2012). Distribution of microbial biomass and potential for anaerobic respiration in hanford site 300 area subsurface sediment. Appl. Environ. Microbiol. 78, 759–767 10.1128/AEM.07404-11 22138990 PMC3264105

[B32] Loewenstein Y. Portugaly E. Fromer M. Linial M. (2008). Efficient algorithms for accurate hierarchical clustering of huge datasets: tackling the entire protein space. Bioinformatics 24, i41–i49 10.1093/bioinformatics/btn174 18586742 PMC2718652

[B33] Loman N. J. Misra R. V. Dallman T. J. Constantinidou C. Gharbia S. E. Wain J. (2012). Performance comparison of benchtop high-throughput sequencing platforms. Nat. Biotechnol. 30, 434–439 10.1038/nbt.2198 22522955

[B34] McDonald D. Clemente J. C. Kuczynski J. Rideout J. R. Stombaugh J. Wendel D. (2012). The Biological Observation Matrix (BIOM) format or: how I learned to stop worrying and love the ome-ome. Gigascience 1, 7 10.1186/2047-217X-1-7 23587224 PMC3626512

[B35] Myers G. (1999). A fast bit-vector algorithm for approximate string matching based on dynamic programming. J. ACM 46, 395–415 10.1145/316542.316550

[B36] Needleman S. B. Wunsch C. D. (1970). A general method applicable to the search for similarities in the amino acid sequence of two proteins. J. Mol. Biol. 48, 443–453 10.1016/0022-2836(70)90057-4 5420325

[B37] Oakley B. B. Carbonero F. Dowd S. E. Hawkins R. J. Purdy K. J. (2012). Contrasting patterns of niche partitioning between two anaerobic terminal oxidizers of organic matter. ISME J. 6, 905–914 10.1038/ismej.2011.165 22113373 PMC3329114

[B38] Oakley B. B. Carbonero F. van der Gast C. J. Hawkins R. J. Purdy K. J. (2010). Evolutionary divergence and biogeography of sympatric niche-differentiated bacterial populations. ISME J. 4, 488–497 10.1038/ismej.2009.146 20054357

[B39] Philippot L. Hallin S. (2006). Molecular analysis of soil denitrifying bacteria, in Molecular Approaches to Soil, Rhizosphere and Plant Microorganism Analysis, eds Cooper J. E. Rao J. R. (Cambridge, MA: CABI Publishing), 146–164

[B40] Poly F. Monrozier L. J. Bally R. (2001). Improvement in the RFLP procedure for studying the diversity of nifH genes in communities of nitrogen fixers in soil. Res. Microbiol. 152, 95–103 10.1016/S0923-2508(00)01172-4 11281330

[B41] Price M. N. Dehal P. S. Arkin A. P. (2010). FastTree 2 – approximately maximum-likelihood trees for large alignments. PLoS ONE 5:e9490 10.1371/journal.pone.0009490 20224823 PMC2835736

[B42] Punta M. Coggill P. C. Eberhardt R. Y. Mistry J. Tate J. Boursnell C. (2012). The Pfam protein families database. Nucl. Acids Res. 40, D290–D301 10.1093/nar/gkr1065 22127870 PMC3245129

[B43] R Core Team. (2012). R: a Language and Environment for Statistical Computing. Vienna: R Foundation for Statistical Computing ISBN 3-900051-07-0. Available online at: www.R-project.org/

[B44] Reeder J. Knight R. (2009). The ‘rare biosphere’: a reality check. Nat. Methods 6, 636–637 10.1038/nmeth0909-636 19718016

[B45] Santos S. R. Ochman H. (2004). Identification and phylogenetic sorting of bacterial lineages with universally conserved genes and proteins. Environ. Microbiol. 6, 754–759 10.1111/j.1462-2920.2004.00617.x 15186354

[B46] Schloss P. D. Westcott S. L. Ryabin T. Hall J. R. Hartmann M. Hollister E. B. (2009). Introducing mothur: open-source, platform-independent, community-supported software for describing and comparing microbial communities. Appl. Environ. Microbiol. 75, 7537–7541 10.1128/AEM.01541-09 19801464 PMC2786419

[B47] Spain A. M. Peacock A. D. Istok J. D. Elshahed M. S. Najar F. Z. Roe B. A. (2007). Identification and isolation of a *Castellaniella* species important during biostimulation of an acidic nitrate- and uranium-contaminated aquifer. Appl. Environ. Microbiol. 73, 4892–4904 10.1128/AEM.00331-07 17557842 PMC1951013

[B48] Stackebrandt E. Ebers J. (2006). Taxonomic parameters revisited: tarnished gold standards. Microbiol. Today 33, 152–155

[B49] Stackebrandt E. Frederiksen W. Garrity G. M. Grimont P. A. Kämpfer P. Maiden M. C. (2002). Report of the ad hoc committee for the re-evaluation of the species definition in bacteriology. Int. J. Syst. Evol. Microbiol. 52(Pt 3), 1043–1047 10.1099/ijs.0.02360-0 12054223

[B50] Stedtfeld R. D. Baushke S. Tourlousse D. Chai B. Cole J. R. Hashsham S. A. (2007). Multiplex approach for screening genetic markers of microbial indicators. Water Environ. Res. 79, 260–269 10.2175/106143007X181378 17469657

[B51] Stres B. Murovec B. (2008). Differences in melting temperatures of degenerated oligonucleotides targeting nitrous oxid reductase (nosz) genes. Acta Argic. Slov. 92, 75–82

[B52] Stres B. Murovec B. (2009). New primer combinations with comparable melting temperatures detecting highest numbers of nosz sequences from sequence databases. Acta Argic. Slov. 94, 139–142

[B53] Suenaga H. (2012). Targeted metagenomics: a high-resolution metagenomics approach for specific gene clusters in complex microbial communities. Environ. Microbiol. 14, 13–22 10.1111/j.1462-2920.2011.02438.x 21366818

[B54] Uniprot Consortium. (2010). Ongoing and future developments at the universal protein resource. Nucl. Acids Res. 39, D214–D219 10.1093/nar/gkq1020 21051339 PMC3013648

[B55] Vital M. Penton C. R. Wang Q. Young V. B. Antonopoulos D. A. Sogin M. L. (2013). A gene-targeted approach to investigate the intestinal butyrate-producing bacterial community. Microbiome 1, 8 10.1186/2049-2618-1-8 PMC4126176 24451334

[B56] Wang Q. Quensen J. F. Fish J. A. Lee T. K. Sun Y. Tiedje J. M. (2013). Ecological patterns of *nifH* genes in four terrestrial climatic zones explored with targeted metagenomics using FrameBot, a new informatics tool. mBio. 4:e00592-13 10.1128/mBio.00592-13 24045641 PMC3781835

[B57] Weisman D. Yasuda M. Bowen J. L. (2013). FunFrame: functional gene ecological analysis pipeline. Bioinformatics 29, 1212–1214 10.1093/bioinformatics/btt123 23511542

[B58] Wertz S. Dandie C. E. Goyer C. Trevors J. T. Patten C. L. (2009). Diversity of *nirK* denitrifying genes and transcripts in an agricultural soil. Appl. Environ. Microbiol. 75, 7365–7377 10.1128/AEM.01588-09 19801455 PMC2786405

[B59] Zhang Y. Sun Y. (2011). HMM-FRAME: accurate protein domain classification for metagenomic sequences containing frames. BMC Bioinform. 12:198 10.1186/1471-2105-12-198 21609463 PMC3115854

